# Cognitive conversion disorder (functional cognitive disorder) – what’s new?

**DOI:** 10.1192/j.eurpsy.2021.498

**Published:** 2021-08-13

**Authors:** J. Morais, S. Fonseca

**Affiliations:** Psychiatry, Centro Hospitalar Universitário de São João, Porto, Portugal

**Keywords:** Functional, Cognitive, conversion

## Abstract

**Introduction:**

Some patients present with significant subjective cognitive symptoms, sometimes interfering with day-to-day live, that are not compatible with any recognizable psychiatric, neurodegenerative or systemic condition. Recent studies have proposed that these patients can be diagnosed with Conversion Disorder (Subtype Cognitive), also known as Functional Cognitive Disorder (FCD). This is a relatively recent concept, that still lacks consensus.

**Objectives:**

Review the current state of knowledge regarding prevalence, diagnosis criteria, core clinical features and proposed treatment of Functional Cognitive Disorder.

**Methods:**

Bibliographic review of the literature published in English in the last 5 years, in the databases Pubmed, PsycINFO and Cochrane. The keywords used were: Functional Cognitive Disorder; Cognition; Conversion Disorder. A review of the titles and abstracts of the resulting articles was made, and selected according to their relevance to the study.

**Results:**

Ten articles related to prevalence, diagnosis, clinical associations and treatment of Functional Cognitive Disorder were selected, of which two were systematic reviews, three descriptive studies, three cross sectional clinical studies of memory clinics attendants, one cohort prospective study and one article was a case series report.

**Conclusions:**

The prevalence of FCD is estimated between 11.6% and 56% of patients presenting to memory clinics. However, the prevalence of FCD is hindered by the lack of consensus regarding its definition. Recently, Ball et al proposed a definition in line with the DSM-5 definition of Conversion Disorder with emphasis on positive criteria with the identification of positive evidence of internal inconsistency. Treatment discussion is still limited, and the approach is similar to other conversion disorders.

